# Environmental factors in gastric carcinogenesis and preventive intervention strategies

**DOI:** 10.1186/s41021-025-00328-w

**Published:** 2025-03-05

**Authors:** Yuzhi Tan, Juntaro Matsuzaki, Yoshimasa Saito, Hidekazu Suzuki

**Affiliations:** 1 Division of Pharmacotherapeutics, Keio University Faculty of Pharmacy, 1-5-30 Shibakoen, Minato-ku, Tokyo, 105-8512 Japan; 2 Department of Gastroenterology and Hepatology, Department of Internal Medicine, Tokai University School of Medicine, 143 Shimokasuya, Isehara, 259-1193 Kanagawa Japan

**Keywords:** Gastric cancer, Prevention, Microbiome, Extracellular vesicles

## Abstract

Gastric cancer, a significant global health concern, arises from a complex interplay of genetic and environmental factors. *Helicobacter pylori* (*H. pylori*) infection is a major risk factor that can be mitigated through eradication strategies. Epstein-Barr virus (EBV) infection causes a distinct subtype of gastric cancer called EBV-associated gastric cancer. The gastric microbiome, a dynamic ecosystem, is also involved in carcinogenesis, particularly dysbiosis and specific bacterial species such as *Streptococcus anginosus*. Long-term use of proton pump inhibitors and potassium-competitive acid blockers also increases the risk of gastric cancer, whereas non-steroidal anti-inflammatory drugs including aspirin may have a protective effect. Smoking significantly increases the risk, and cessation can reduce it. Dietary factors such as high intake of salt, processed meats, and red meat may increase the risk, whereas a diet rich in fruits and vegetables may be protective. Extracellular vesicles, which are small membrane-bound structures released by cells, modulate the tumor microenvironment and may serve as biomarkers for risk stratification and as therapeutic targets in gastric cancer. This review highlights the multifaceted etiology of gastric cancer and its risk factors and emphasizes the importance of a multi-pronged approach to prevention including *H. pylori* eradication and modification of lifestyle factors, as well as the potential of microbiome-based and EV-based interventions. Further research is needed to refine risk stratification and to develop personalized prevention strategies.

## Background

**Fig. 1:**
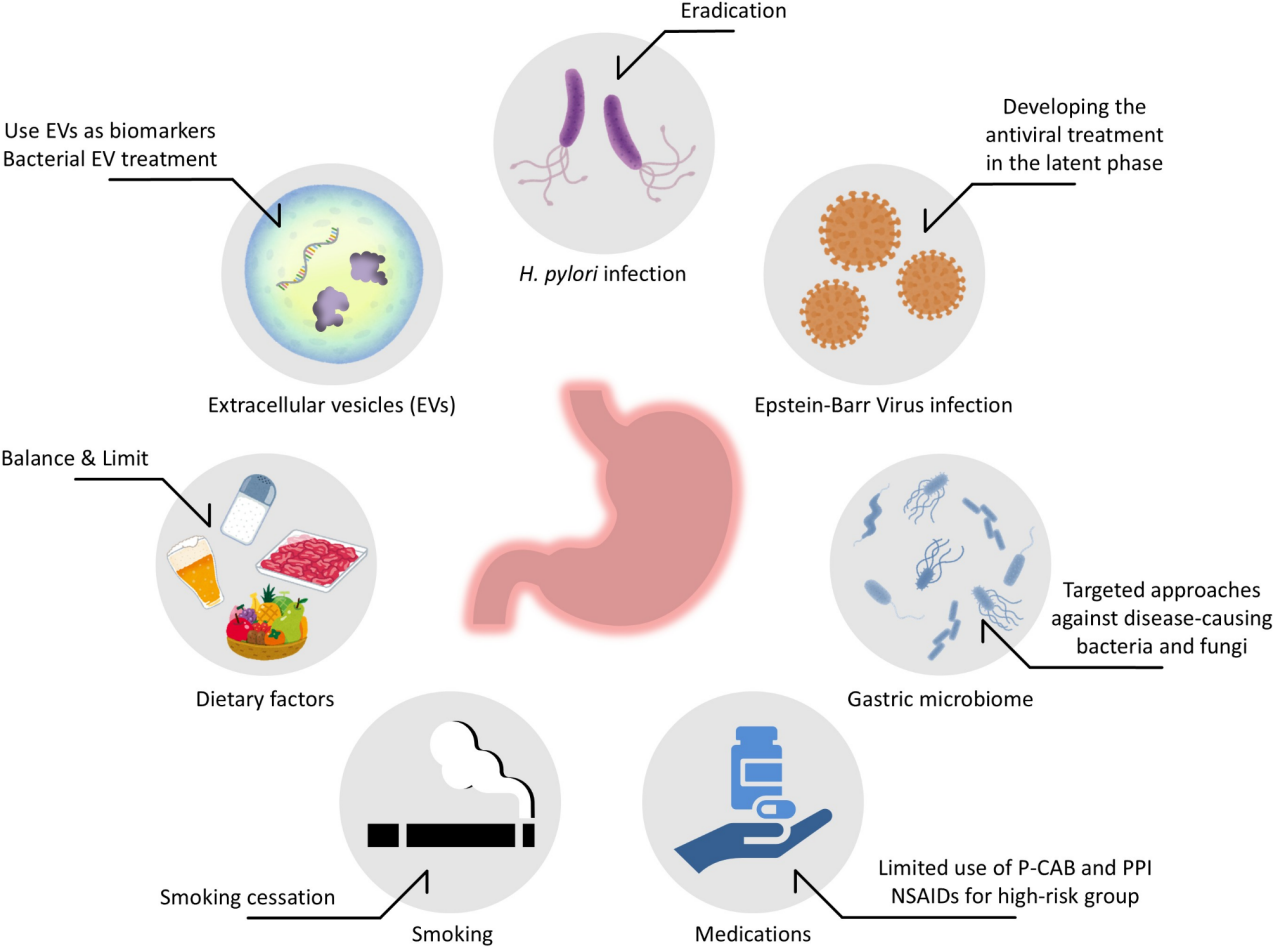
Potential interventions for the prevention of gastric cancer

According to GLOBOCAN 2022, gastric cancer is the fifth most common malignancy and the sixth leading cause of cancer-related death worldwide [1]. The causal relationship between *Helicobacter pylori* (*H. pylori)* infection and the development of gastric cancer has been demonstrated [2]. However, only 2.9% of patients with *H. pylori* infection developed gastric cancer over a mean follow-up period of 7.8 years [3]. A recent study showed that germline pathogenic variants in nine genes (APC, ATM, BRCA1, BRCA2, CDH1, MLH1, MSH2, MSH6, and PALB2) are associated with the risk of gastric cancer [4]. At 85 years of age, persons with *H. pylori* infection and one pathogenic variant had a higher cumulative risk of gastric cancer than noncarriers infected with *H. pylori* (45.5% [95% confidence interval (CI), 20.7–62.6] vs. 14.4% [95% CI, 12.2–16.6]). Epidemiological data and basic research suggest that various environmental factors, such as medications, lifestyle, and commensal bacteria increase the risk of gastric carcinogenesis. Although germline pathogenicity cannot be a direct interventional target for gastric cancer prevention, environmental factors can be modified as preemptive medicine. This review summarizes current data on environmental factors associated with the risk of gastric cancer and discusses potential interventions for preventing gastric cancer (Fig. 1).

## Main text

### *H. pylori* infection

*H. pylori* is a gram-negative bacterium classified as a Group 1 carcinogen by the International Agency for Research on Cancer (IARC) in 1994 [5]. *H. pylori* is considered a causative factor in gastric cancer, and several underlying mechanisms have been proposed, among them the oncogenic protein cytotoxin-associated gene A (CagA) [6–8]. *H. pylori* produces CagA and delivers it into the gastric epithelial cell cytoplasm via a type IV transporter. Once inside the host cell, CagA undergoes tyrosine phosphorylation at the EPIYA motifs, which are short amino acid sequences containing the EPIYA (Glu-Pro-Ile-Tyr-Ala) sequence [9]. The combination and number of EPIYA motifs can influence the biological activity of CagA, which may explain the difference in the prevalence of gastric cancer between East Asian and Western countries [10]. In addition, CagA/PAR1b interaction subverts nuclear translocation of BRCA1 by inhibiting PAR1b-mediated BRCA1 phosphorylation, leading to BRCAness-mediated genome instability [11].

A systematic review and meta-regression analysis that investigated the trends in *H. pylori* infection rates in Japan over nearly a century found a clear birth-cohort pattern in the prevalence of *H. pylori* infection, with infection rates decreasing significantly in successive generations [12]. The predicted prevalence of *H. pylori* infection was 60.9% for those born in 1910, but only 6.6% for those born in 2000. Another survey conducted between 2010 and 2019 in China revealed that the prevalence of *H. pylori* infection was lowest in adults aged 18–24 years (39.71%), gradually increased in the middle-aged group (40–59 years, 51.92%) and peaked in the older group (60–94 years, 53.24%) [13]. As the prevalence of *H. pylori* infection increases with age and is accompanied by a corresponding rise in the incidence rate of gastric cancer, eradication of *H. pylori* remains a critical preventive strategy. Eradicating *H. pylori* decreases the incidence of gastric cancer [14–18]. A recent systematic review and meta-analysis showed that among 2480 individuals in whom *H pylori* was eradicated, 163 (6.57%) developed metachronous gastric cancer, compared with 176 (12.73%) among 1383 persistently infected individuals. The pooled risk ratio of metachronous gastric cancer in these studies was 0.46 [95% CI, 0.37–0.57] [19]. These results support the implementation of eradication programs as a public health strategy [20].

However, the abnormalities in DNA methylation patterns caused by the chronic inflammation induced by *H. pylori* infection may not disappear after *H. pylori* eradication. Many studies have suggested that these abnormalities are a risk factor for gastric cancer [21, 22]. The levels of DNA methylation in the gastric mucosa after eradication remain high and are reported to be useful for predicting gastric cancer and extensive precancerous lesions [23].

### Epstein-barr virus infection

Epstein-Barr virus (EBV), also known as human herpesvirus 4 (HHV-4), is a member of the herpesvirus family. EBV is one of the most common viruses in humans. It is estimated that > 90% of the world’s population has been infected with EBV by adulthood. Primary EBV infection often occurs in childhood and is usually asymptomatic or presents with mild symptoms [24]. In adolescents and young adults, it can cause infectious mononucleosis, which is characterized by fever, sore throat, swollen lymph nodes, and fatigue. Once EBV enters the body, it establishes a lifelong latent infection in the host.

EBV is also classified by the IARC as a Group 1 human carcinogen and is associated with lymphomas, including Burkitt lymphoma, Hodgkin lymphoma, post-transplant lymphoproliferative disorder, and NK/T-cell lymphoma. It is also associated with epithelial cancers such as nasopharyngeal carcinoma and gastric cancer, as well as with autoimmune disorders such as multiple sclerosis [25]. EBV infects the epithelial cells of the gastric mucosa, leading to clonal expansion of infected cells. EBV is associated with a distinct subtype of gastric cancer known as EBV-associated gastric cancer (EBVaGC), which is characterized by recurrent PIK3CA mutations, extreme DNA hypermethylation, and amplification of JAK2, PD-L1, and PD-L2 [26]. EBVaGC accounts for approximately 10% of gastric cancers [27] and is more frequent in men and younger individuals. It occurs predominantly in the proximal stomach and is associated with a diffuse adenocarcinoma histology [28]. In EBVaGC, EBV-encoded microRNAs (miRNAs) alter apoptotic gene expression apoptosis and play important regulatory roles [29]. EBV infection has been suggested to be casually linked to the development of lymphoepithelioma-like gastric carcinoma, which constitutes 1–5% of all gastric carcinomas [30]. A recent systematic review and meta-analyses reported that the prevalence rate of EBV infection in patients with lymphoepithelioma-like gastric carcinoma is significantly higher than that in conventional adenocarcinoma. Additionally, the prevalence rate in patients from East Asia (82.5% [95% CI, 75.2–88.0%]) is higher than in other regions (29.5% [95% CI, 6.4–72.0%]) [31].

There is no specific strategy to reduce the risk of EBVaGC because there is no treatment against EBV in the latent phase. Research is ongoing to develop more effective treatments and potential vaccines to prevent EBVaGC, as well as to achieve a deeper understanding of the role of EBV infection in EBVaGC [25].

### Gastric microbiome

Recent advances in DNA sequencing technology confirmed the existence of a diverse gastric microbiome, which plays a crucial role in gastric cancer development. For example, the duration of gastric lesion development is longer in germ-free INS-GAS mice (gastrin-overexpressing transgenic mice) than in specific pathogen-free (SPF) INS-GAS mice [32]. Co-infection of germ-free INS-GAS mice with *H. pylori* and three other intestinal bacteria (*Clostridium* species, *Lactobacillus murinus*, and *Bacteroides* species) resulted in gastric neoplasia formation, similar to the effect of *H. pylori* infection under SPF conditions [33]. Therefore, the gastric microbiome is strongly associated with gastric carcinogenesis. *H. pylori* infection disrupts the composition of the gastric microbiome, and eradication of *H. pylori* partially improves dysbiosis [34], suggesting that modulation of the gastric microbiome is one of the mechanisms by which *H. pylori* promotes gastric cancer development.

Cross-sectional studies have described the differences in the bacterial composition of the stomach between patients with gastric cancer and those with atrophic gastritis [35]. However, whether the altered bacteria are pathogenic remains unclear. The importance of *Streptococcus anginosus* (*S. anginosus*) in gastric carcinogenesis was recently suggested. A multicenter study in China reported significant enrichment in *S. anginosus* and *S. constellatus* in gastric tissues and feces from patients with intraepithelial neoplasia and early and advanced gastric cancer [36]. The gastritis-promoting effect of *S. anginosus* was confirmed in mice. TMPC, a surface protein in *S. anginosus*, interacts with the Annexin A2 receptor on gastric epithelial cells; it mediates attachment and colonization of *S. anginosus* and induces MAPK activation [37]. This mechanism may explain the involvement of *S. anginosus* in gastric tumorigenesis.

In addition to bacteria, fungi may also be involved in gastric carcinogenesis. Candida species found in gastric cancer samples were linked to the expression of pro-inflammatory immune pathways and correlated with poor survival [38]. Both antibiotics and antifungals have a broad effect on the gut microbiome and affect disease outcomes and clinical efficacy. Novel targeted and tailored approaches against disease-causing bacteria and fungi should be explored in future research and in clinical practice [39]. Although probiotics may reduce the risk of gastric cancer, there is no clear supporting evidence. A systematic analysis and meta-analysis reported that probiotics can reduce gastric cancer-related inflammation more effectively by increasing the levels of CD4^+^ T cells and reducing the levels of IL-6 in the stomach [40].

### Medications

The association between the incidence of gastric cancer and medication history has been discussed for decades. Gastric acid suppressants, such as potassium-competitive acid blockers (P-CAB) and proton pump inhibitors (PPIs), may increase the risk of gastric cancer. P-CAB and PPIs are commonly used in the treatment of acid-related conditions, such as gastroesophageal reflux disease and peptic ulcers. Long-term suppression of gastric acid secretion by P-CAB/PPI use can drastically change the gastric microbiome [41]. The overgrowth of pathobionts can induce inflammatory responses in the stomach, potentially leading to the development of gastric cancer. In a UK population-based cohort, the risk of gastric cancer was 45% higher in PPI users than in histamine-2 receptor antagonist (H2RA) users (hazard ratio [HR] 1.45, 95% CI, 1.06–1.98) [42]. In a Korean population database study, the adjusted HR for gastric cancer in PPI users was 1.15 (95% CI, 1.06–1.25) compared with non-PPI users. Long-term PPI use after *H. pylori* eradication is associated with increased gastric cancer risk with a dose-dependent effect. Gastric cancer-related mortality does not differ significantly between PPI users and non-users [43]. A Japanese population database study found that the use of P-CABs is associated with a higher gastric cancer risk than the use of H2RA among *H. pylori*-eradicated patients (matched HR, 1.92; 95% CI, 1.13–3.25). Longer duration of P-CAB use and higher doses are significantly associated with a higher incidence of gastric cancer [44]. The studies above compared gastric cancer incidence rates, not cancer-related mortality rates. P-CAB users may generally undergo esophagogastroduodenoscopy more frequently than H2RA users, and this bias may have influenced the results. In addition, a randomized controlled trial has revealed that five-year use of P-CAB and PPI showed no difference in the incidence of gastric cancer [45]. Thus, P-CAB/PPIs should not be considered unduly dangerous, but patients and healthcare providers need to weigh the benefits and risks of long-term PPI use. PPIs should be used at the lowest effective dose and for the shortest duration necessary to manage symptoms, especially in individuals with *H. pylori* infection history [46].

The role of non-steroidal anti-inflammatory drugs (NSAIDs), including aspirin, in cancer prevention has been well described for gastrointestinal cancers [47]. Two large prospective U.S. cohort studies reported that the risk of gastric adenocarcinoma is 48% lower in regular aspirin users (at least twice a week) than in non-users (multivariable HR, 0.52; 95% CI, 0.37–0.73) among women. This protective effect increases with longer duration of use and higher doses. However, no significant association was found between aspirin use and gastric adenocarcinoma risk in men [48]. In a large Korean nationwide cohort study and a systematic review, researchers found that both aspirin and statin use were significantly associated with a reduced risk of developing gastric cancer. Metformin, however, was not associated with gastric cancer risk. The study found that statins have a strong protective effect against gastric cancer in individuals with diabetes. The meta-analysis further supported these findings, demonstrating the highest effect size for statin, followed by aspirin and metformin in reducing gastric cancer risk [49]. A Japanese multicenter retrospective cohort study investigated the association between NSAIDs and the risk of gastric cancer in patients who used PPIs after *H. pylori* eradication. The study found that the risk of developing gastric cancer was 60% lower in NSAID users than in non-NSAID users. This protective effect increased with both higher doses and longer durations of NSAID use. There were no gastric cancer cases among patients who used cyclooxygenase-2 (COX-2) inhibitor NSAIDs. These findings suggest that NSAIDs have a chemopreventive effect and may reduce the risk of gastric cancer in individuals who use PPIs after *H. pylori* eradication [50].

### Smoking

Smoking is a well-established risk factor for gastric cancer, and studies show that smokers have a higher risk of developing gastric cancer than non-smokers. The risk increases with the number of cigarettes smoked per day and the duration of smoking. A large-scale pooled analysis of 23 epidemiological studies examined the association between cigarette smoking and gastric cancer risk [51]. The study found that the risk of gastric cancer is significantly higher in ever smokers than in never smokers. Current smokers have a higher risk than former smokers, and the risk increases with both the number of cigarettes smoked per day and the duration of smoking. The risk of gastric cancer decreases with increasing time since quitting smoking, reaching levels similar to those in never smokers approximately 10 years after cessation. The study also found that the risk is slightly higher for cardia gastric cancer than non-cardia gastric cancer. These findings provide strong evidence supporting the detrimental effect of cigarette smoking on gastric cancer risk and highlight the potential benefits of smoking cessation.

### Dietary factors

Dietary factors are associated with the risk of developing gastric cancer. High intake of salt and salt-preserved foods, such as pickled vegetables and salted fish, increases the risk of gastric cancer. A high salt content can damage the stomach lining and promote the growth of *H. pylori*, which is linked to gastric cancer. A meta-analysis showed that a preference for salty taste, consistent use of table salt, and high intake of salt-preserved foods are associated with increased risk of gastric cancer, whereas total sodium intake was not associated with increased risk [39]. The same group demonstrated an increased risk of gastric cancer associated with the consumption of processed meat and red meat in a meta-analysis [52]. Consumption of processed meats (such as sausages and ham) and red meats is linked to a higher risk of gastric cancer. These foods often contain nitrates and nitrites, which can form carcinogenic compounds in the stomach. In a systematic review and meta-analysis, Liu et al. demonstrated that increased consumption of processed meat increases the risk of gastric cancer, which is supported by moderate-quality evidence [53]. The same study suggested a positive correlation between gastric cancer risk and alcohol consumption, supported by high-quality evidence [53]. However, the increased gastric cancer risk due to alcohol consumption may differ between men and women [54]. A diet rich in fruits and vegetables is generally associated with a lower risk of gastric cancer, although the correlation between fruit and vegetable intake and gastric cancer risk is weak [53, 55]. These findings highlight the importance of dietary choices in the prevention of gastric cancer.

### Extracellular vesicles

Extracellular vesicles (EVs) are membrane-bound nanovesicles released by cells into the extracellular environment [56, 57]. They play a crucial role in intercellular communication by transporting proteins, lipids, and nucleic acids between cells. EVs are implicated in various aspects of gastric cancer development, including immune responses and inflammation.

EVs may serve as potential targets for the prevention of gastric cancer. The contents of EVs released from gastric epithelial cells are modified during carcinogenesis [58, 59]. Atrophic gastritis, a precancerous condition for gastric cancer, is associated with specific miRNAs in serum EVs. miR-122-5p is considered as a potential biomarker for chronic atrophic gastritis [60]. A systematic review and meta-analysis that explored the diagnostic utility of miRNAs and long non-coding RNAs (lncRNA) within EVs suggested that the contents of EVs could serve as diagnostic biomarkers in gastric cancer [61]. The contents of EVs vary during to the progression of gastric cancer and may be secreted to either promote or inhibit its progression. miR-122-5p is downregulated in gastric cancer and suppresses gastric tumor proliferation and metastasis by targeting GIT1 [62], suggesting that promoting miR-122-5p function could suppress chronic atrophic gastritis and potentially inhibit the development of gastric cancer.

Serum EVs from patients with stage I/II gastric cancer contain elevated levels of one lncRNA (GClnc1), suggesting its potential as a biomarker for distinguishing patients with early gastric cancer from healthy individuals and those with precancerous conditions such as chronic atrophic gastritis and intestinal metaplasia [63]. Sun et al. performed RNA sequencing and enrichment analysis of gastric cancer cells subjected to shRNA-mediated GClnc1 knockdown and found that gene sets associated with the exacerbation of gastric cancer were upregulated [64]. These findings suggest that GClnc1 is involved in the transition from precancerous lesions to early-stage gastric cancer. miR-181a-5p downregulates ATM, whose pathogenic variants interact with *H. pylori* infection to increase additively the risk of gastric cancer [4, 65]. A lncRNA (CCAT1) downregulates miR-181a-5p, and serum EV CCAT1 levels have been reported to be significantly higher in patients with gastric cancer than in healthy control subjects [66]. Gastric cancer cells may maintain intracellular levels of miR-181a-5p and suppress ATM expression by secreting CCAT1 into EVs. EVs derived from cells infected with *H. pylori* contain elevated levels of CagA, PD-L1, and HSP60 [67, 68], suggesting the potential utility of these proteins as diagnostic biomarkers of *H. pylori* infection, precancerous conditions, and gastric cancer.

Certain EVs derived from gut microbiota may help to suppress gastric inflammation. *Enterococcus faecium* and its EVs protect against ethanol-induced gastric injury in rats by reducing inflammation, enhancing antioxidative functions, and improving mucosal glycoprotein production [69]. Similarly, EVs and cell-free supernatant derived from *Lactobacillus crispatus* mitigate *H. pylori*-induced inflammation in human gastric adenocarcinoma cells by modulating the expression of pro- and anti-inflammatory cytokines. EVs are thus potential therapeutic agents against *H. pylori*-triggered inflammation [70], and because of their anti-inflammatory effects, EVs may serve as a strategy for cancer prevention.

## Conclusions

Traditionally, clinicians have relied on the severity of gastric mucosal atrophy to predict the risk of gastric carcinogenesis. However, assessment of the tumor microenvironment and lifestyle choices is necessary for risk stratification. Proper risk stratification will enable the optimization of surveillance strategies for gastric cancer. Currently, the intervals between esophagogastroduodenoscopies are not individualized. Potentially effective prevention strategies should be provided only for high-risk groups. Promising strategies should be verified comprehensively and prospectively, although obtaining evidence supporting the efficacy of treatments in reducing mortality could require a long time. Well-designed prospective studies are necessary to develop optimal strategies for preventing gastric cancer.

## Data Availability

No datasets were generated or analysed during the current study.
